# Sjögren's Disease-Associated Renal Tubular Acidosis: An Integrated Immuno-Tubular Model of Tubular Dysfunction

**DOI:** 10.7150/ijms.136917

**Published:** 2026-07-30

**Authors:** Chien-Lin Lu, Chia-Chao Wu, Te-Chao Fang, Kuo-Cheng Lu

**Affiliations:** 1 Division of Nephrology, Department of Internal Medicine, Fu Jen Catholic University Hospital, Fu Jen Catholic University, New Taipei City 24352, Taiwan.; 2 School of Medicine, College of Medicine, Fu Jen Catholic University, New Taipei City 24205, Taiwan.; 3 Division of Nephrology, Department of Internal Medicine, Tri-Service General Hospital, National Defense Medical Center, Taipei 11490, Taiwan.; 4 Division of Nephrology, Department of Internal Medicine, School of Medicine, College of Medicine, Taipei Medical University, Taipei 11031, Taiwan.; 5 Division of Nephrology, Department of Internal Medicine, Taipei Medical University Hospital, Taipei Medical University, Taipei 11031, Taiwan.; 6 Taipei Medical University-Research Center of Urology and Kidney, Taipei Medical University, Taipei 11031, Taiwan.; 7 Division of Nephrology, Department of Medicine, Taipei Tzu Chi Hospital, Buddhist Tzu Chi Medical Foundation, New Taipei City 23142, Taiwan.

**Keywords:** acidosis, citrate, hypocitraturia, renal tubular acidosis, Sjögren's disease, tubulointerstitial nephritis

## Abstract

Sjögren's disease (SjD) is traditionally viewed as an autoimmune exocrinopathy; however, renal involvement—particularly renal tubular acidosis (RTA)—is an underrecognized extraglandular manifestation that often precedes classical sicca symptoms and contributes to diagnostic delay. Distal RTA (dRTA) represents the dominant phenotype and arises from immune-mediated injury to α-intercalated cells, leading to disruption of key acidification components, including vacuolar H^+^-ATPase (V-ATPase), carbonic anhydrase II (CAII), and anion exchanger 1 (AE1). Beyond impaired distal proton secretion, systemic acidosis is proposed to induce adaptive responses in the proximal tubule, based largely on general renal physiology rather than SjD-specific data. Intracellular acidification may enhance sodium-dependent dicarboxylate cotransporter 1 (NaDC-1)-mediated citrate reabsorption and mitochondrial citrate metabolism. These processes may reduce urinary citrate excretion (hypocitraturia) and promote a lithogenic milieu characterized by calcium phosphate supersaturation. This integrated distal-proximal axis provides a plausible mechanistic link between tubular dysfunction and clinical manifestations such as nephrolithiasis, nephrocalcinosis, and hypokalemic paralysis. Incomplete dRTA further expands the disease spectrum and is frequently overlooked despite its clinical relevance. Current management is centered on alkali and potassium replacement, which correct metabolic abnormalities but do not address underlying immune-mediated injury. Treatment response is stage-dependent and often limited once fibrosis is established. This review proposes an integrated immuno-tubular framework that links clinical phenotype, pathogenesis, and therapeutic strategy, and highlights the need for early detection, biomarker-driven risk stratification—particularly using hypocitraturia—and incorporation of renal-specific endpoints to enable mechanism-based and precision-guided care.

## 1. Introduction

Sjögren's disease (SjD) is traditionally conceptualized as an autoimmune exocrinopathy defined by sicca symptoms; however, this gland-centric view does not adequately capture a subset of patients in whom extraglandular manifestations predominate 1, 2. In this review, the term "Sjögren's disease" is used in place of the older "Sjögren's syndrome" to emphasize its systemic autoimmune nature rather than a symptom-defined syndrome. In clinical practice, renal tubular acidosis (RTA)—particularly distal RTA (dRTA)—often presents with hypokalemic paralysis, nephrolithiasis, or nephrocalcinosis, frequently in the absence of overt sicca features 3, 4. In a compilation of 440 published case reports and small case series of SjD-associated RTA (rather than a systematically ascertained cohort), only 7.7% had a prior diagnosis of SjD, whereas hypokalemic paralysis was the initial presentation in 63.6% 3. Because this dataset aggregates individually published cases rather than a prospectively ascertained population, these figures likely overstate the true frequency of clinically dramatic presentations such as hypokalemic paralysis due to reporting/publication bias, and are best interpreted as characterizing the published case literature rather than the general SjD-RTA population. As a result, patients are commonly evaluated within nephrological or neurological pathways without recognition of an underlying systemic autoimmune disorder, leading to diagnostic delay 5, 6. Ongoing tubulointerstitial nephritis (TIN) may therefore progress to irreversible fibrosis before disease-modifying therapy is initiated 1, 2.

Despite its clinical importance, SjD-associated RTA remains conceptually fragmented. Clinical studies largely emphasize electrolyte disturbances and their downstream complications. In contrast, mechanistic work focuses on immune-mediated disruption of the distal acidification apparatus, including carbonic anhydrase II (CAII), vacuolar H^+^-ATPase (V-ATPase), and anion exchanger 1 (AE1) 2, 3. These perspectives are rarely integrated into a framework that connects immune injury to impaired urinary acidification and clinical phenotypes. Subclinical forms such as incomplete dRTA—rare frequently overlooked despite their association with hypocitraturia and increased stone risk 7-9. In a single-center Brazilian cohort of 42 patients, hypocitraturia predicted dRTA with 100% sensitivity and 91% specificity 7; while this finding highlights that reliance on overt acidosis may underestimate disease burden, it derives from a small cohort and awaits external validation before its diagnostic performance can be generalized.

Therapeutic strategies remain largely empirical. Alkali replacement corrects metabolic abnormalities but does not address the underlying immune-mediated injury, and the role of immunosuppression in restoring tubular function is not well defined 2, 4. In addition, renal-specific endpoints—such as urinary acidification capacity, tubular biomarkers, transporter expression, and nephrocalcinosis progression—are seldom incorporated into clinical studies, limiting mechanistic assessment of treatment response 10, 11.

This review proposes an integrated immuno-tubular framework for SjD-associated RTA that links clinical presentation, immunopathogenesis, and therapeutic strategy. RTA is conceptualized as a continuum from subclinical tubular dysfunction (incomplete dRTA) to overt metabolic acidosis, with emphasis on emerging biomarkers for earlier detection and risk stratification. This approach aims to support a shift from empiric management toward mechanism-based, precision-guided care.

## 2. Classification of RTA in Sjögren's Disease: Clinical Relevance and Phenotypic Spectrum

RTA in SjD encompasses multiple physiological defects, but their clinical distribution is highly uneven, with dRTA accounting for the vast majority of cases. Although all classical subtypes can occur, most patients present with distal tubular dysfunction, whereas proximal or hyperkalemic forms are uncommon and typically reflect broader or secondary processes.

### 2.1 Distal RTA (Type 1): The Dominant Phenotype

dRTA represents the principal renal phenotype in SjD and reflects impaired acid secretion by α-intercalated cells in the collecting duct. Proton secretion depends on V-ATPase and basolateral bicarbonate transport via AE1 12, 13. In SjD, dysfunction of this system occurs in the setting of autoimmune TIN, with evidence of immune-mediated disruption of V-ATPase, CAII, and AE1 2, 14.

The biochemical profile is characterized by hyperchloremic normal anion-gap metabolic acidosis with an inappropriately high urine pH (>5.5) despite systemic acidemia 3. Impaired ammonium excretion is typically reflected by a positive urinary anion gap (UAG) 13. Hypokalemia is common and may be severe, resulting from increased distal potassium loss and occasionally presenting as hypokalemic paralysis 3, 15.

Persistently alkaline urine, together with hypercalciuria and hypocitraturia, promotes calcium phosphate precipitation, leading to nephrolithiasis and nephrocalcinosis. Chronic acidosis further contributes to bone demineralization and osteomalacia 16. Clinically, dRTA frequently precedes classical sicca symptoms and is a major driver of delayed diagnosis in SjD 15, 17.

### 2.2 Proximal RTA (Type 2): Rare and Syndromic Presentation

Proximal RTA (pRTA) is uncommon in SjD and usually occurs as part of generalized proximal tubular dysfunction rather than as an isolated defect. It reflects impaired bicarbonate reabsorption in the proximal tubule, leading to bicarbonate wasting and normal anion-gap metabolic acidosis 18, 19. In SjD, proximal involvement is most often associated with autoimmune TIN affecting proximal tubular segments 20.

Additional mechanisms may include reduced expression of endocytic receptors such as megalin and cubilin, potentially driven by Th17-mediated inflammation and ectopic germinal center formation 21. Clinically, pRTA typically presents as Fanconi syndrome, with urinary losses of bicarbonate, phosphate, glucose, uric acid, and amino acids 1, 22. Associated findings—such as hypophosphatemia, hypouricemia, and normoglycemic glycosuria—serve as key diagnostic clues 1, 20. Compared with dRTA, proximal involvement is considerably less frequent and usually reflects more diffuse tubular injury rather than a dominant disease phenotype.

### 2.3 Type 4 RTA and Mixed Forms: Uncommon but Relevant Variants

Type 4 RTA is rare in SjD and is characterized by hyperchloremic normal anion-gap metabolic acidosis with hyperkalemia, typically resulting from aldosterone deficiency or resistance 13, 23. Reduced aldosterone activity impairs sodium reabsorption via the epithelial sodium channel (ENaC), diminishing the lumen-negative potential and reducing distal hydrogen and potassium secretion 13. Consistent with this mechanism, urine pH in type 4 RTA is typically appropriately low (< 5.5), reflecting preserved distal acidification despite reduced net acid excretion.

When present, type 4 RTA in SjD often reflects additional contributing conditions, including TIN, diabetes, obstructive uropathy, or medication effects such as renin-angiotensin system inhibitors or potassium-sparing diuretics 24, 25. Large case series confirm its low prevalence, with only a small proportion of SjD-associated RTA cases demonstrating a hyperkalemic phenotype 3.

Mixed forms, combining distal and proximal defects, are more frequently recognized and usually indicate more extensive tubulointerstitial involvement 13. These mixed forms reflect combined defects in both distal acid secretion and proximal bicarbonate handling and are therefore best interpreted as markers of more extensive tubulointerstitial injury rather than a distinct disease category. Clinically, the coexistence of hypokalemia with features of proximal tubular dysfunction or variable acidification capacity should prompt consideration of mixed RTA.

### 2.4 Incomplete Distal RTA: A Subclinical and Early Disease Form

Incomplete dRTA represents a subclinical defect in urinary acidification in which serum bicarbonate remains normal at baseline but maximal urinary acidification is impaired 26, 27. Diagnosis requires provocative testing, such as ammonium chloride loading or the furosemide-fludrocortisone test, demonstrating failure to lower urine pH below approximately 5.3-5.5 27. Ammonium chloride loading remains the diagnostic gold standard but is frequently poorly tolerated, commonly causing nausea, vomiting, and abdominal discomfort, and it induces a systemic acid load that warrants caution in patients with hepatic impairment or baseline acidosis 28, 29. The furosemide-fludrocortisone test avoids this acid load and is associated with substantially fewer gastrointestinal side effects, making it more feasible for routine outpatient use 28. However, its diagnostic performance is test- and population-dependent: in a prospective cohort of kidney stone formers, furosemide-fludrocortisone testing showed 77% sensitivity, 85% specificity, and a high negative predictive value (98%) but a low positive predictive value (30%) against ammonium chloride, supporting its role in screening or exclusion rather than confirmation 30. Notably, in primary Sjögren's disease specifically, furosemide-fludrocortisone testing performed less well by every diagnostic metric against ammonium chloride: sensitivity and specificity were 65% and 68%, respectively (compared with 77% and 85% in the stone-former cohort above), with a correspondingly lower negative predictive value (82% vs. 98%). Positive predictive value was numerically higher (46% vs. 30%), a difference attributable at least in part to the substantially higher prevalence of incomplete dRTA in the SjD cohort (25%) than in the stone-former cohort (8%), which mathematically raises PPV and lowers NPV independent of test accuracy. The authors concluded that the furosemide-fludrocortisone test should not replace ammonium chloride loading for confirmatory diagnosis in this population 9. In routine clinical practice, the furosemide-fludrocortisone test is therefore reasonably used as an initial, better-tolerated screening tool, with ammonium chloride loading reserved for confirmatory testing when the diagnosis remains uncertain. Rather than a discrete entity, incomplete dRTA is best understood as part of a continuum of distal acidification capacity reflecting partial α-intercalated cell dysfunction 31, 32.

Despite the absence of overt acidosis, incomplete dRTA is clinically relevant. Patients often present with nephrolithiasis or nephrocalcinosis driven by persistently alkaline urine and hypocitraturia 13, 33. In autoimmune settings, including SjD, it may represent early tubular injury preceding overt dRTA 2. Recognition is particularly important in patients with unexplained recurrent calcium stones, as early intervention may prevent progression. These features underscore incomplete dRTA as an underrecognized early disease phenotype and a potential target for early detection.

The clinical, biochemical, and phenotypic distinctions among RTA subtypes in SjD are summarized in Table 1.

## 3. Pathogenesis of RTA in Sjögren's Disease: An Integrated Immuno-Tubular Model

**Figure 1:**
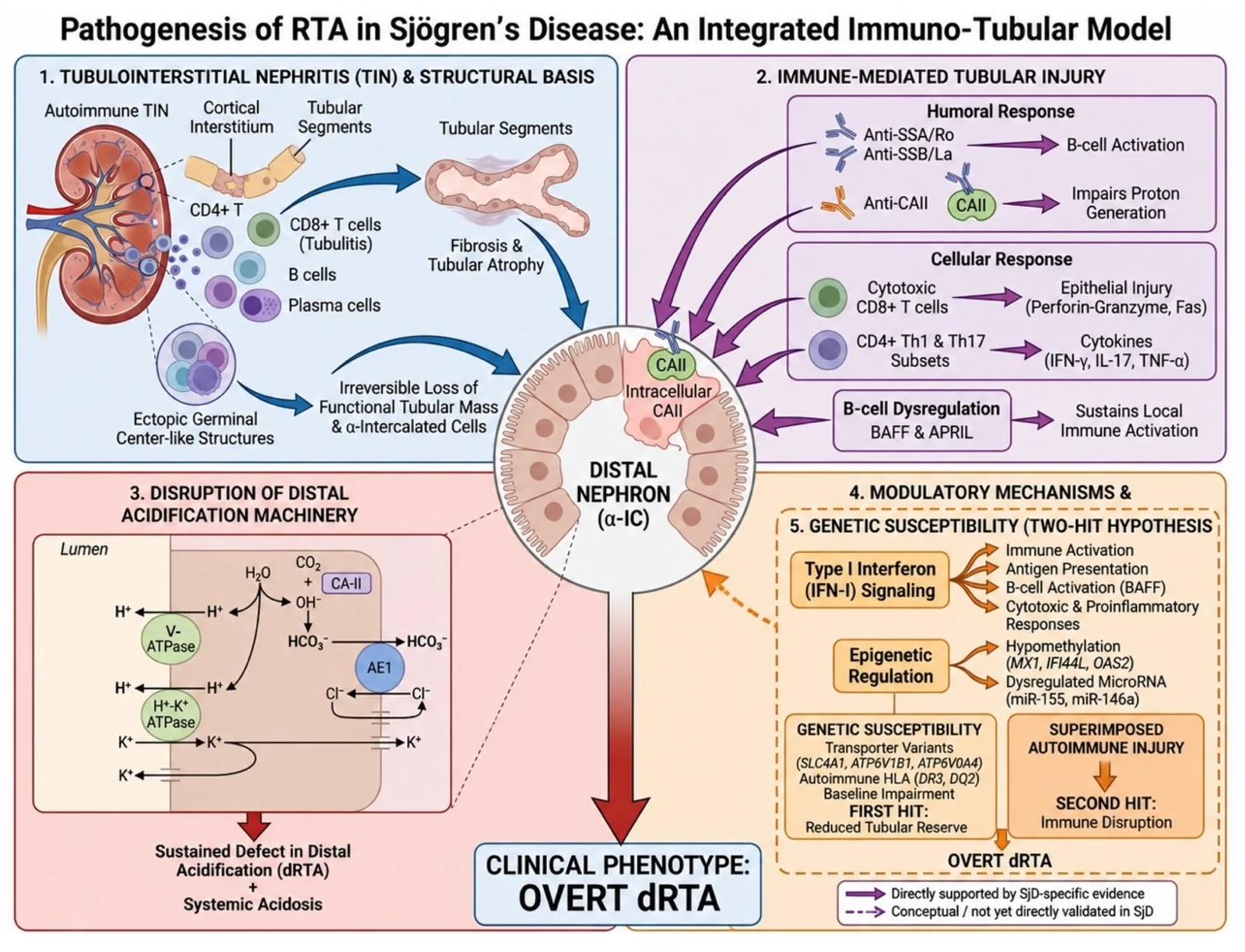
Integrated immuno-tubular model of renal tubular acidosis in Sjögren's disease. Renal tubular acidosis (RTA) in Sjögren's disease (SjD) arises from converging structural, immune, molecular, and genetic mechanisms targeting the distal nephron. (1) Tubulointerstitial nephritis (TIN) with loss of α-intercalated cells; (2) Immune-mediated tubular injury; (3) Disruption of distal acidification machinery (V-ATPase, CAII, and AE1); (4) Type I interferon and epigenetic amplification; (5) Genetic susceptibility via a two-hit model. Solid borders/arrows denote components (1-4) directly supported by SjD-specific evidence; the dashed border/arrow denotes the two-hit genetic susceptibility model, which remains conceptual and has not been directly validated in SjD. Abbreviations: AE1, anion exchanger 1; α-IC, α-intercalated cell; APRIL, a proliferation-inducing ligand; BAFF, B-cell activating factor; CAII, carbonic anhydrase II; dRTA, distal renal tubular acidosis; H^+^-K^+^-ATPase, non-gastric H^+^,K^+^-ATPase; IFN-I, type I interferon; RTA, renal tubular acidosis; SjD, Sjögren's disease; TIN, tubulointerstitial nephritis; V-ATPase, vacuolar H^+^-ATPase.

RTA in SjD arises from immune-mediated injury targeting distal tubular cells, with disruption of acidification machinery representing the functional endpoint. Rather than a single mechanism, dRTA reflects a coordinated network of immunological, molecular, and structural processes targeting α-intercalated cells in the distal nephron. These integrated immuno-tubular mechanisms are summarized in Figure 1.

### 3.1 Tubulointerstitial Nephritis as the Structural Substrate

Autoimmune TIN forms the structural basis of renal involvement in SjD and directly impairs tubular architecture and function 1, 34. Inflammatory infiltrates are composed predominantly of CD4^+^ T lymphocytes, with contributions from CD8⁺ T cells, B cells, and plasma cells 20. CD8^+^ T cells frequently infiltrate tubular epithelium (tubulitis), supporting direct cytotoxic injury, while B-cell aggregates may form ectopic germinal center-like structures, indicating localized immune activation 35.

Inflammation is typically distributed in the cortical interstitium surrounding tubular segments involved in urinary acidification 20. Persistent injury promotes interstitial fibrosis and tubular atrophy, resulting in irreversible loss of functional tubular mass 1, 34. Loss of viable α-intercalated cells progressively limits acidification capacity and underlies the development of persistent dRTA in advanced disease 3, 36.

### 3.2 Immune-Mediated Tubular Injury

Tubular injury in SjD is driven by coordinated humoral and cellular immune responses targeting renal epithelial cells 1, 20. Autoantibodies such as anti-SSA/Ro and anti-SSB/La reflect B-cell activation and are associated with renal involvement 37. Among these, anti-CAII antibodies are mechanistically relevant, as they impair intracellular proton generation and are associated with reduced urinary acidification and increased urine pH 38.

Cellular immunity amplifies this process. Cytotoxic CD8^+^ T cells induce epithelial injury through perforin-granzyme and Fas-mediated pathways, while CD4^+^ T-cell subsets, including Th1 and Th17 cells, promote inflammation via cytokines such as interferon-γ, interleukin-17, and tumor necrosis factor-α 39, 40. In parallel, B-cell dysregulation through B-cell activating factor (BAFF) and a proliferation-inducing ligand (APRIL) sustains local immune activation and supports ectopic lymphoid structures 41, 42. These combined mechanisms result in persistent epithelial injury and progressive tubular dysfunction.

### 3.3 Disruption of Distal Acidification Machinery

dRTA reflects failure of coordinated proton secretion by α-intercalated cells in the collecting duct. This process depends on apical V-ATPase, intracellular CAII, and basolateral AE1, which together maintain proton secretion and bicarbonate transport 43, 44. Disruption of any component reduces net acid excretion and impairs urinary acidification in the setting of systemic acidosis 32, 45. These processes converge on α-intercalated cells in the distal nephron, which represent the functional locus of acidification failure in SjD-associated dRTA.

In SjD, immune-mediated injury affects multiple elements of this system. Reduced or mislocalized V-ATPase impairs apical proton secretion, while inhibition of CAII limits intracellular proton generation 43. AE1 dysfunction disrupts basolateral bicarbonate extrusion, leading to intracellular alkalinization and secondary V-ATPase mislocalization 46. In some cases, selective loss of AE1 or combined transporter defects further reduce the population of functional α-intercalated cells 47. The net effect is a sustained defect in distal acidification that defines the clinical phenotype of dRTA.

**Figure 2:**
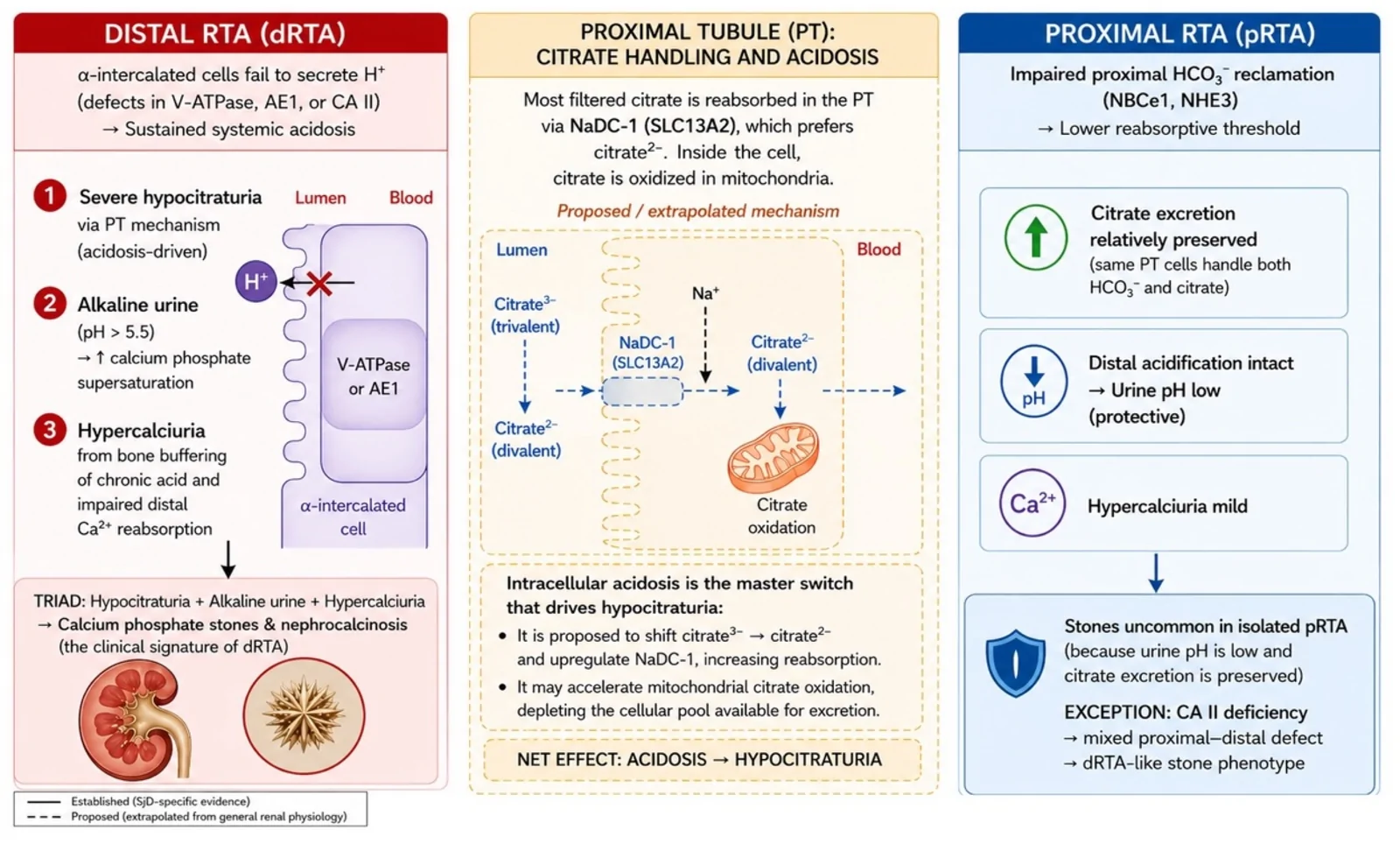
Proposed model of distal acidification failure and acidosis-driven proximal citrate handling in Sjögren's disease-associated renal tubular acidosis. Schematic of the distal acidification defect (V-ATPase, CAII, AE1) and its proposed downstream link to proximal citrate handling and hypocitraturia. Solid lines/borders denote the distal pathway, directly supported by SjD-specific evidence; dashed lines/borders denote the proximal citrate-handling pathway, extrapolated from general renal physiology and not yet directly confirmed in SjD. Abbreviations: AE1, anion exchanger 1; CAII, carbonic anhydrase II; dRTA, distal renal tubular acidosis; NaDC-1, sodium-dependent dicarboxylate cotransporter 1; NBCe1, electrogenic sodium bicarbonate cotransporter 1; NHE3, sodium/hydrogen exchanger 3; PT, proximal tubule; pRTA, proximal renal tubular acidosis; V-ATPase, vacuolar H+-ATPase.

However, impaired distal acidification alone does not fully account for the characteristic lithogenic phenotype observed in SjD-associated dRTA. The proximal contribution to this phenotype should be regarded as a proposed, rather than an established, mechanism: direct SjD-specific evidence for NaDC-1 regulation and mitochondrial citrate flux is currently lacking, although pH-dependent proximal citrate handling is well supported by general renal physiology studies. On this basis, systemic acidosis is proposed to induce adaptive proximal tubular responses that may increase citrate reabsorption and reduce urinary citrate excretion, resulting in hypocitraturia (Figure 2) 7, 45, 48. Beyond this proposed NaDC-1-mediated pathway, hypocitraturia in dRTA is also compounded by concurrent hypokalemia, which independently promotes proximal citrate reabsorption through intracellular acidification and increased NHE3-mediated hydrogen secretion, irrespective of systemic pH, and by dietary factors such as high animal-protein or low-alkali intake 49. Hypercalciuria, the other key contributor to the lithogenic phenotype, arises predominantly from chronic metabolic acidosis itself: sustained buffering of the systemic acid load mobilizes calcium and phosphate from bone, while acidosis independently reduces distal tubular calcium reabsorption, together increasing urinary calcium excretion largely independent of dietary calcium intake 50. Together with alkaline urine and hypercalciuria, hypocitraturia favors calcium phosphate supersaturation and provides a plausible, though not yet directly confirmed, link between distal tubular dysfunction and nephrolithiasis or nephrocalcinosis 7, 48.

### 3.4 Modulatory Mechanisms: Interferon and Epigenetic Regulation

Type I interferon (IFN-I) signaling is a central amplifier of immune activation in SjD, with widespread upregulation of interferon-stimulated genes in both immune and epithelial compartments 51, 52. IFN-I enhances antigen presentation, promotes B-cell activation via BAFF, and increases cytotoxic and proinflammatory responses 53. In tubular epithelial cells, interferon signaling induces cytokine production and cell death pathways, contributing to sustained tubulointerstitial injury 54. These pathways act as upstream modulators that amplify and sustain immune-mediated tubular injury.

Epigenetic mechanisms reinforce this inflammatory state. Hypomethylation of interferon-responsive genes—including MX1, IFI44L, and OAS2—enhances transcriptional responsiveness to IFN signaling 51, 55, 56. Dysregulated microRNA expression, particularly increased miR-155 and altered miR-146a, modulates pathways such as nuclear factor κB (NF-κB) and Janus kinase/signal transducer and activator of transcription (JAK/STAT), influencing cytokine production and cell survival 57, 58. These processes sustain chronic inflammation and perpetuate tubular injury.

### 3.5 Genetic Susceptibility and the Two-Hit Hypothesis

Genetic factors may plausibly contribute to susceptibility to RTA in SjD by affecting both tubular transport and immune regulation; however, this remains a conceptual framework rather than an established disease mechanism, and direct validation in SjD is currently lacking. Variants in genes encoding acidification transporters—such as solute carrier family 4 member 1 (SLC4A1; AE1), ATP6V1B1, and ATP6V0A4—impair proton secretion and can produce subclinical defects in tubular acidification 45, 59-61. In parallel, autoimmune susceptibility is associated with HLA haplotypes, particularly HLA-DR3 and DQ2, which predispose to dysregulated immune responses 62, 63. This baseline impairment may be conceptualized as reduced tubular reserve, which predisposes to overt dysfunction upon immune-mediated injury, although this link has not been directly demonstrated in SjD-associated RTA.

On this basis, a two-hit model has been proposed, in which reduced tubular reserve is unmasked by superimposed autoimmune injury: baseline impairment of acidification capacity would represent the first hit, while immune-mediated disruption of transport proteins would constitute the second, leading to overt dRTA once functional compensation is exceeded. This model is illustrated with a dashed border in Figure 1 to distinguish it from the directly evidenced mechanisms (1-4), as no study has yet demonstrated a direct interaction between transporter gene variants and autoimmune tubular injury in SjD-associated dRTA. Direct validation of this model in SjD remains limited, and integration of genetic and immunological data will be required to define its clinical relevance.

## 4. Clinical Presentation and Diagnostic Approach to RTA in Sjögren's Disease

RTA in SjD spans a wide clinical spectrum, from incidental biochemical abnormalities to life-threatening complications. Diagnosis requires integration of clinical presentation, biochemical findings, and autoimmune evaluation to facilitate timely recognition.

### 4.1 Clinical Presentation: From Subclinical Disease to Acute Manifestations

RTA in SjD most commonly presents through hypokalemia rather than acid-base abnormalities. As noted above, in the 440-case published compilation, hypokalemic paralysis was recorded as the presenting feature in 63.6% of published cases, with respiratory paralysis in 8.4% 3; given the case-report-based nature of this dataset, these frequencies should not be equated with true population prevalence. Clinical manifestations include fatigue, muscle weakness, acute flaccid quadriparesis, and, in severe cases, respiratory failure requiring ventilatory support 64, 65. These manifestations typically occur in distal RTA with marked hypokalemia and normal anion-gap metabolic acidosis. Hypokalemic paralysis may represent the first manifestation of previously unrecognized SjD, particularly in the absence of sicca symptoms 6, 17.

Chronic distal RTA produces renal and skeletal complications through persistently alkaline urine, hypercalciuria, and hypocitraturia. This environment favors calcium phosphate stone formation and nephrocalcinosis, presenting as renal colic, hematuria, recurrent stones, or incidental imaging findings 45. Nephrolithiasis and nephrocalcinosis are associated with renal dysfunction in SjD 66. Long-standing acidosis contributes to bone demineralization, osteomalacia, and chronic bone pain 45, 67. The presence of unexplained distal RTA, hypokalemic paralysis, nephrocalcinosis, or recurrent calcium stones should prompt evaluation for SjD.

### 4.2 Biochemical Confirmation of Renal Tubular Acidosis

Diagnosis is typically established by demonstrating hyperchloremic normal anion-gap metabolic acidosis with impaired renal acid handling. Key laboratory findings include reduced serum bicarbonate in the setting of preserved glomerular filtration, while potassium levels help distinguish subtypes—hypokalemia in distal and proximal RTA, and hyperkalemia in type 4 RTA 68. Alternative causes, including extrarenal bicarbonate loss and advanced chronic kidney disease, should be excluded before confirming a tubular origin 69, 70.

Urinary acidification assessment is central to evaluation. In distal RTA, urine pH remains inappropriately elevated (> 5.3-5.5) despite systemic acidemia 45. Reduced ammonium excretion is inferred from a positive UAG, whereas a negative UAG suggests extrarenal bicarbonate loss 68. When results are inconclusive, the urine osmolal gap can provide an estimate of ammonium excretion 71.

The fractional excretion of bicarbonate (FEHCO_3_) helps differentiate subtypes. Values < 5% support distal RTA, whereas values > 10-15% indicate proximal RTA 68, 72. Additional urinary findings aid in identifying underlying tubular disorders: low-molecular-weight proteinuria, glycosuria, phosphaturia, and aminoaciduria suggest Fanconi syndrome, whereas isolated tubular abnormalities without significant glomerular proteinuria are more typical of SjD-associated TIN 45.

### 4.3 Etiological Evaluation: Autoimmunity, Imaging, and Differential Diagnosis

After biochemical confirmation, evaluation focuses on identifying SjD and excluding alternative causes. Serological testing is central. Anti-SSA/Ro and anti-SSB/La antibodies are frequently detected and correlate with renal involvement and disease severity 73, 74. Additional findings—including antinuclear antibodies (ANA), rheumatoid factor, and polyclonal hypergammaglobulinemia—support systemic autoimmune activity 74, 75. Complement consumption (low C3/C4) and cryoglobulinemia are associated with renal involvement and increased lymphoma risk 66, 74. Anti-CAII antibodies provide mechanistic support for tubular dysfunction 38.

Imaging and histology further refine diagnosis. Ultrasonography or computed tomography is typically used to detect nephrolithiasis and nephrocalcinosis, which are strongly linked to renal dysfunction in SjD 66, 73. Renal biopsy is reserved for cases with diagnostic uncertainty, unexplained decline in kidney function, or when immunosuppressive therapy is considered. Histopathology typically demonstrates plasma cell-rich TIN and allows assessment of inflammatory activity and fibrosis 74, 76. Biopsy findings directly inform therapeutic decisions: active, cellular TIN supports initiation of immunosuppressive therapy, whereas predominant interstitial fibrosis and tubular atrophy indicate a lower likelihood of treatment response and favor continued metabolic (alkali-based) management alone (see Section 5.1).

A structured differential diagnosis is essential. Drug-induced RTA should be excluded, particularly with exposure to amphotericin B, tenofovir, or ifosfamide 45, 77. Other autoimmune diseases, including systemic lupus erythematosus and immunoglobulin G4-related disease (IgG4-related disease), may produce similar tubulointerstitial pathology and should be distinguished based on serology and histology 74. In younger patients or those with a suggestive family history, hereditary distal RTA should be considered, with genetic testing for SLC4A1 and V-ATPase subunits when appropriate 59, 78. A summary of key distinguishing features across these differential diagnoses is provided in Table 2.

## 5. Therapeutic Strategies for RTA in Sjögren's Disease: A Clinical Framework

Management of RTA in SjD requires simultaneous control of metabolic abnormalities and assessment of underlying immune activity. While alkali therapy is generally required in patients with overt dRTA, immunomodulatory treatment is reserved for selected cases with active TIN or progressive renal involvement. Current practice is largely based on observational data, with limited randomized evidence.

### 5.1 Principles of Management

Management generally involves two parallel priorities: correction of metabolic derangements and identification of patients who may benefit from immunosuppression. Alkali and potassium replacement correct acidosis and hypokalemia, reduce recurrent weakness, and limit long-term complications including nephrolithiasis, nephrocalcinosis, and bone disease 1, 2. Maintenance therapy is often required long term in most patients, typically at 1-2 mEq/kg/day in dRTA 79. In acute presentations such as hypokalemic paralysis or respiratory failure, prompt potassium and alkali replacement leads to rapid clinical improvement 14, 80.

Immunosuppressive therapy is considered in patients with biopsy-proven active TIN, declining kidney function, or persistent tubular dysfunction despite adequate metabolic control 2, 76. Corticosteroids are the most commonly used initial agent, with mycophenolate mofetil, azathioprine, cyclophosphamide, or rituximab used in selected cases with severe, relapsing, or mixed renal involvement 1, 42, 76. Treatment response is strongly stage-dependent: active inflammatory TIN may improve, whereas established fibrosis is typically associated with persistent acidification defects and ongoing alkali requirement 10, 81.

### 5.2 Metabolic Correction: Alkali and Potassium Management

Alkali replacement corrects systemic acidosis and remains the cornerstone of therapy in all patients with overt dRTA. The treatment target is normalization of serum bicarbonate, generally ≥ 22 mmol/L 82. Sodium bicarbonate and potassium citrate are both effective; however, potassium-containing alkali is generally preferred in patients with hypokalemia or stone disease because it corrects potassium deficit, increases urinary citrate, and reduces calcium stone risk 13, 83. Sodium-based alkali may increase urinary calcium and should be used cautiously in patients with nephrolithiasis or volume-sensitive conditions 13.

Potassium management depends on severity. Chronic hypokalemia is typically managed with oral potassium salts, whereas severe hypokalemia or hypokalemic paralysis requires intravenous replacement with cardiac monitoring 82. Correction of acidosis is necessary for sustained potassium control, as persistent acidosis promotes renal potassium wasting 13, 83.

Long-term therapy improves biochemical control, reduces stone risk, and stabilizes nephrocalcinosis 84, 85. Monitoring should include serum bicarbonate and potassium, urinary calcium and citrate, kidney function, and treatment adherence, as incomplete correction is common in routine practice 82.

### 5.3 Immunomodulatory Therapy: Indications and Treatment Hierarchy

No formal treatment guidelines exist for SjD-associated RTA. The treatment hierarchy described below is therefore derived from observational cohorts, case series, and extrapolation from the management of TIN in other autoimmune contexts, rather than from formal, evidence-graded recommendations, and should be interpreted accordingly. Direct, controlled evidence linking specific immunosuppressive agents to renal tubular outcomes is limited: reported benefit is largely confined to case series and individual reports of improved urinary acidification, with corticosteroids showing the most consistent benefit when initiated during active inflammation, and no agent, including rituximab, has been evaluated in controlled trials with renal tubular function as a defined endpoint.

Immunosuppression is reserved for patients with evidence of active TIN, particularly when associated with declining kidney function or persistent tubular dysfunction. Treatment decisions are individualized based on disease activity and reversibility 86, 87. Earlier treatment appears to be associated with better renal recovery, whereas delayed therapy in the presence of fibrosis is linked to incomplete response 87.

Corticosteroids are the most commonly used initial agent and are reported to be most effective during the inflammatory phase 86, 88. In patients with relapse, steroid dependence, or incomplete response, steroid-sparing agents such as mycophenolate mofetil or azathioprine are commonly used, based on case-series experience 89. Hydroxychloroquine is often used for systemic disease control but has not been shown to directly improve tubular acidification 90. Calcineurin inhibitors have been reported in refractory cases but require caution due to nephrotoxicity, and supporting data are limited to small series 86. Biologic therapies, including rituximab or tumor necrosis factor-α inhibitors, have shown benefit in individual cases or small series, although their role in SjD-associated RTA remains uncertain and is not supported by controlled trial data 91, 92. The available evidence on renal outcomes and reversibility of tubular dysfunction for each agent is summarized in Table 3.

### 5.4 Advanced and Emerging Therapies: Limited Evidence

Targeted therapies have been explored in refractory or severe disease, but renal-specific data remain limited. Rituximab has been reported in case-based and small series to improve renal involvement in individual cases, including recovery of urinary acidification 93. However, randomized trials in SjD have shown inconsistent systemic benefit, and renal tubular outcomes have not been systematically evaluated 94, 95. At present, rituximab is best considered an off-label option in selected refractory cases.

Other targeted approaches, including B-cell activating factor-directed therapy and Janus kinase inhibition, are supported by mechanistic rationale but lack renal outcome data. Agents targeting the BAFF axis (e.g., belimumab, ianalumab, telitacicept) have shown effects on systemic disease activity but their effects on tubular endpoints have not been evaluated. Similarly, Janus kinase inhibitors may suppress interferon-driven inflammation but remain investigational in SjD-associated RTA 96, 97. Their clinical role will depend on future studies incorporating renal-specific endpoints.

### 5.5 Long-Term Management and Monitoring

Long-term management focuses on sustained metabolic control, prevention of complications, and monitoring of disease progression. Treatment targets include normalization of serum bicarbonate and potassium and control of urinary calcium excretion, although adequate control is achieved in only approximately half of patients in cohort studies 82, 84. Lifelong alkali therapy is often required, with dose adjustment based on disease stability and stone activity 13, 98.

Nephrocalcinosis is common and may affect up to 88% of patients, often in association with nephrolithiasis and declining kidney function 84. Chronic acidosis contributes to bone demineralization and fracture risk, which can be mitigated with sustained correction and appropriate vitamin D supplementation 99, 100. Standard chronic kidney disease management should be applied in patients with progressive renal involvement 1.

Long-term follow-up may also include surveillance for lymphoma in selected patients with persistent systemic activity or B-cell-driven disease features 98, 101. Treatment response is best assessed using combined biochemical and clinical parameters, as structural damage due to fibrosis often limits reversibility despite adequate therapy.

## 6. Clinical Integration: A Mechanism-Informed Diagnostic and Management Pathway

**Figure 3:**
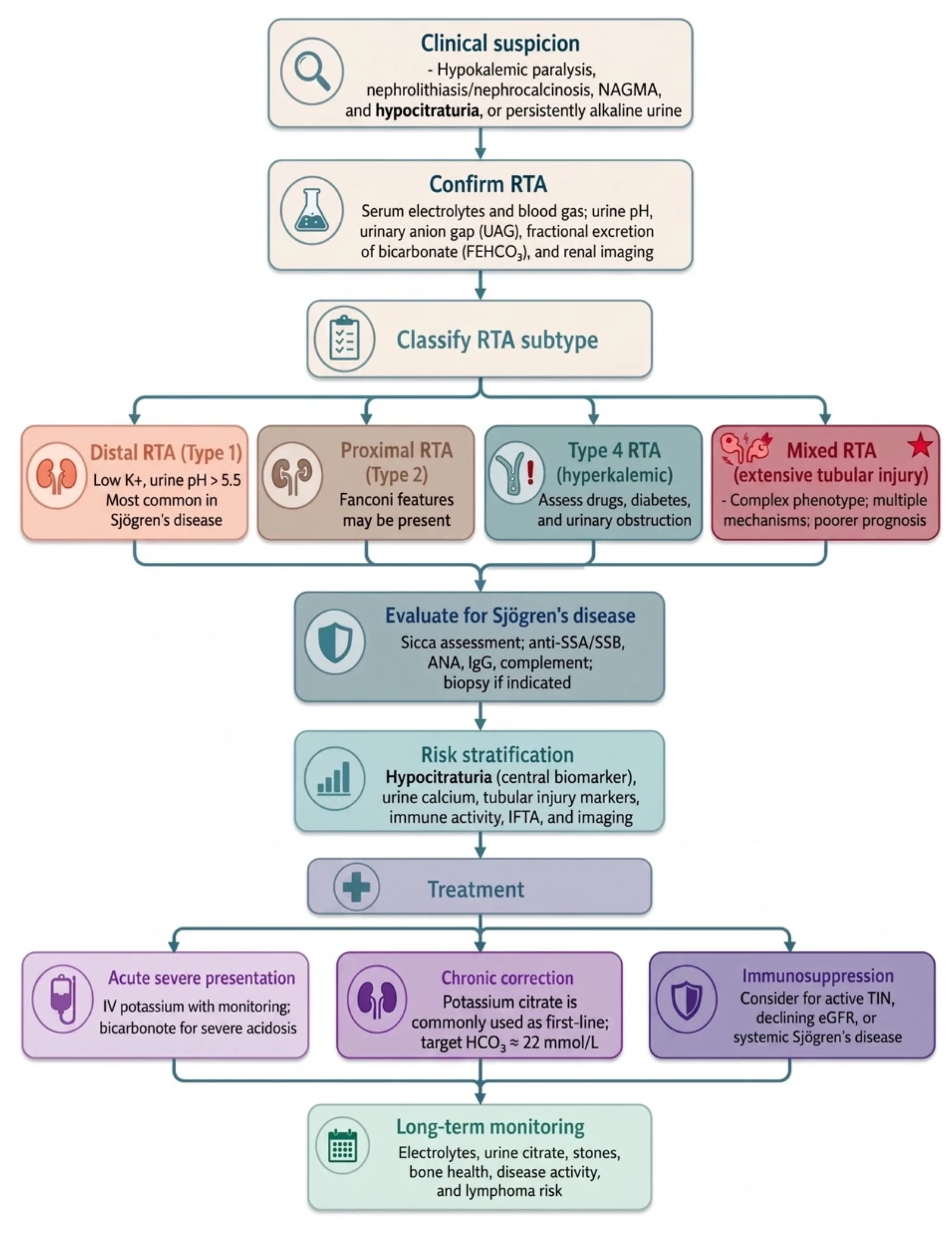
** Mechanism-informed clinical pathway for the diagnosis and management of Sjögren's disease-associated renal tubular acidosis.** Stepwise pathway from clinical suspicion through biochemical confirmation, RTA subtype classification, serologic/histologic evaluation for SjD, risk stratification (hypocitraturia as a lithogenic risk biomarker), and management (metabolic correction with or without immunosuppression). Abbreviations: ANA, antinuclear antibody; eGFR, estimated glomerular filtration rate; FEHCO₃, fractional excretion of bicarbonate; HCO₃, bicarbonate; IFTA, interstitial fibrosis and tubular atrophy; IgG, immunoglobulin G; NAGMA, normal anion-gap metabolic acidosis; RTA, renal tubular acidosis; SjD, Sjögren's disease; UAG, urinary anion gap.

The mechanistic framework described above can be translated into a structured clinical pathway for SjD-RTA (Figure 3). Evaluation begins when clinical suspicion is raised by hypokalemic paralysis, recurrent calcium phosphate nephrolithiasis or nephrocalcinosis, unexplained normal anion-gap metabolic acidosis, persistently alkaline urine, or hypocitraturia. Because renal manifestations may precede sicca symptoms, early recognition requires a high index of suspicion across clinical settings. Biochemical confirmation relies on identifying hyperchloremic metabolic acidosis with preserved glomerular filtration, supported by urine pH, urinary anion gap, and, when appropriate, fractional excretion of bicarbonate and imaging findings.

Following confirmation, classification of RTA subtype provides diagnostic direction. dRTA represents the dominant phenotype in SjD, whereas proximal, mixed, and type 4 forms suggest broader or alternative tubular involvement and should prompt evaluation for contributing conditions such as medications, diabetes, or urinary obstruction. Once RTA is established, focused evaluation for underlying SjD is warranted, including serologic testing (anti-SSA/Ro, anti-SSB/La, ANA), immunologic markers, and, when indicated, tissue biopsy. Recognition of the autoimmune context informs both prognosis and treatment selection.

Risk stratification links pathophysiology to clinical outcomes and guides management intensity. Hypocitraturia serves as a clinically useful biomarker, reflecting the combined effects of distal acidification failure and proximal tubular adaptation and associating with lithogenic risk. Management proceeds along two complementary axes: correction of metabolic derangements, typically with potassium-based alkali therapy, and consideration of immunosuppressive treatment in selected patients with active tubulointerstitial inflammation or progressive renal dysfunction.

Long-term management requires integrated monitoring of electrolytes, urinary parameters, stone burden, and systemic disease activity. This pathway provides a practical framework for translating mechanistic insights into individualized, mechanism-informed clinical care.

## 7. Special Clinical Contexts and Unresolved Challenges in Sjögren's Disease-Associated RTA

RTA in SjD is associated with persistent diagnostic and therapeutic challenges, particularly in early recognition, management of special populations, and limited evidence for treatment selection. These issues affect both clinical outcomes and the ability to implement individualized care.

### 7.1 Diagnostic Challenges and Interdisciplinary Gaps

Renal manifestations in SjD often precede systemic diagnosis and may remain unrecognized for years. dRTA frequently presents with hypokalemic paralysis, nephrolithiasis, or nephrocalcinosis, while SjD is only identified later following autoimmune evaluation 6, 48. Renal involvement is well documented to precede, coincide with, or follow sicca symptom onset, though the literature does not converge on a fixed interval: in a large multicenter cohort, renal involvement preceded SjD diagnosis in 17% of affected patients, was identified concurrently with SjD diagnosis in 53%, and developed at a median of 8.7 years after SjD diagnosis in the remaining 30% 102; conversely, an earlier biopsy series found that SjD diagnosis preceded renal manifestations by a median of 5.5 years 103. Nephrolithiasis and nephrocalcinosis are reported in approximately 5-21% of patients across small, single-center cohort series 73, with wide variability attributable to differences in imaging modality and referral patterns; a similar pattern is also frequently described in the published case-report literature, where it often represents the presenting clinical clue 3.

Renal involvement is not the only extraglandular presentation that can precede or occur independently of overt sicca symptoms, and awareness of these additional clues may further support earlier interdisciplinary recognition of SjD. Musculoskeletal symptoms are common and may initially draw attention away from an underlying autoimmune diagnosis: non-erosive polyarthralgia occurs in up to 96% of patients and frank polyarthritis in approximately 16.6% 104. Cutaneous vasculitis, most often manifesting as palpable purpura, is reported in 10-30% of patients and, together with urticarial vasculitis (0.8-21%), frequently co-occurs with anti-SSA/Ro and anti-SSB/La positivity 104. Peripheral neuropathy is similarly informative: pure sensory neuropathy occurs in 40-49% and sensorimotor polyneuropathy in up to 56% of neurologically affected patients, and may itself be the presenting feature in the absence of sicca complaints 104. Hypocomplementemia (low C3 in 10-15%, low C4 in 5-20%) and cryoglobulinemia frequently accompany these presentations and additionally flag increased lymphoma risk 104. Beyond dRTA, other urological manifestations may serve as clues. Interstitial cystitis-like urinary symptoms are more common in SjD than in the general population: a Finnish population-based study found such symptoms up to 15-20 times more frequent among SS patients than controls 105, and a subsequent large Taiwanese nationwide cohort confirmed increased risk of both bladder pain syndrome/interstitial cystitis (HR 2.34) and overactive bladder (HR 1.68) in primary SjD 106. Interstitial cystitis has occasionally been reported as the presenting clue to previously unrecognized SjD rather than a later complication 107, and in the largest biopsy-confirmed case series, bladder involvement was rarely severe enough to cause acute obstructive renal failure 108. Acute presentations such as respiratory failure due to severe hypokalemic paralysis from distal RTA have similarly been reported as the initial manifestation of previously unrecognized SjD—in some cases with no prior sicca symptoms and initial misdiagnosis as a neurologic condition such as Guillain-Barré syndrome 109-111. As with renal involvement, recognizing these atypical presentations requires a high index of suspicion, particularly because even overt sicca symptoms are associated with substantial diagnostic delay in their own right, with a median lag of 115 weeks (over two years) between symptom onset and diagnosis reported in a population-based cohort **112**.

This presentation pattern leads to fragmented care, with patients evaluated in nephrology, rheumatology, dermatology, or neurology settings without consideration of an underlying autoimmune disorder. Delay in diagnosis allows progression from active inflammation to fibrosis, reducing the likelihood of treatment response 74, 113. Early recognition remains challenging and requires consideration of SjD in patients with unexplained dRTA, hypokalemia, or recurrent calcium stones, even in the absence of sicca symptoms 2. Integration of nephrology and rheumatology pathways remains inconsistent, and standardized referral strategies are lacking. Strengthening this interdisciplinary integration will likely require several concrete strategies. Standardized referral criteria could be established so that unexplained dRTA, persistent hypokalemia, or recurrent calcium nephrolithiasis automatically prompts serologic screening (anti-SSA/Ro, anti-SSB/La, ANA) and rheumatology referral, while conversely, rheumatologists managing confirmed SjD could incorporate routine screening for urinary acidification defects (urine pH, urinary anion gap) as part of standard extraglandular surveillance. Joint nephrology-rheumatology clinics or regular multidisciplinary case conferences may facilitate earlier recognition and shared management of biopsy-proven TIN. Embedding structured clinical decision-support prompts into electronic health records—triggered by combinations such as hypokalemia with alkaline urine, or nephrocalcinosis on imaging—could further reduce reliance on individual clinician awareness. Finally, multicenter registries linking nephrology and rheumatology cohorts would support standardized, prospective validation of candidate biomarkers such as hypocitraturia and enable systematic study of referral pathways and their impact on diagnostic delay.

### 7.2 Special Populations: Pregnancy and Pediatric Disease

Pregnancy may unmask or exacerbate dRTA due to physiological changes in renal function and electrolyte handling. Patients may present with hypokalemia, metabolic acidosis, or myopathy, and SjD-associated dRTA is occasionally first recognized during pregnancy 114-116. Management often requires escalation of bicarbonate and potassium supplementation to maintain maternal stability 114, 117. Maternal acidosis and hypokalemia may affect fetal outcomes, and anti-SSA/Ro positivity necessitates monitoring for neonatal lupus and congenital heart block 2, 114. Immunosuppressive therapy should be selected with attention to fetal safety, with prednisone, hydroxychloroquine, or azathioprine preferred when indicated 114.

Pediatric SjD represents a distinct phenotype in which extraglandular manifestations, including RTA and TIN, may predominate. Presentations include hypokalemic paralysis, nephrocalcinosis, nephrolithiasis, nephrogenic diabetes insipidus, and metabolic bone disease 12, 118-121. Chronic acidosis may impair growth and skeletal development. Evaluation should include autoimmune testing in children with unexplained RTA and exclusion of hereditary causes when indicated 82. Management focuses on sustained metabolic correction, with immunosuppressive therapy considered in cases with active inflammatory disease 119, 122.

### 7.3 Incomplete RTA: Screening and Treatment Uncertainty

Incomplete dRTA represents a subclinical defect in urinary acidification without overt metabolic acidosis. Its reported prevalence varies considerably across dedicated SjD cohort studies 3, 7, 9; by contrast, the 440-case published compilation does not report a comparable incomplete-dRTA prevalence figure and is therefore not included in this comparison. For example, prevalence was 25% in a Dutch cohort using the ammonium chloride gold standard 9, 11.3% for combined complete and incomplete dRTA in a Norwegian cohort 123, and under 6% in an Indian prospective cohort 8 and a Brazilian cohort applying different diagnostic criteria 7. This heterogeneity most likely reflects differences in acidification test method, urine pH threshold, and case ascertainment rather than true differences in underlying disease burden, and the figures cited above should be interpreted as approximate rather than precise population prevalence estimates. Diagnosis requires provocative testing, although the furosemide-fludrocortisone test is often used in practice due to better tolerability 2, 9. Persistent urine pH >5.5 and hypocitraturia may identify patients requiring further evaluation 7, 9.

Clinical implications remain uncertain. Patients may develop nephrolithiasis, nephrocalcinosis, and bone disease despite normal serum bicarbonate 9, 31. In the single-center cohort noted above, hypocitraturia showed high sensitivity and specificity for identifying dRTA 7, though this finding has not yet been replicated in an independent SjD cohort. However, progression from incomplete to overt dRTA is variable, and predictors of progression are not well defined 8. Evidence for pharmacologic intervention is limited, and the role of routine treatment remains unclear. On this basis, urinary citrate assessment cannot yet be recommended as a routine screening test in SjD patients without overt acidosis; its current value lies primarily in risk stratification among patients with an established RTA diagnosis or clinical suspicion (e.g., recurrent nephrolithiasis, persistently alkaline urine), rather than as a universal screening tool. Prospective, multi-center studies validating its sensitivity and specificity in unselected SjD populations are needed before broader screening use can be justified.

### 7.4 Therapeutic Uncertainty and Evidence Gaps

Reported prevalence estimates for both subclinical and overt RTA-related complications in SjD should be interpreted with caution. As summarized in Table 4, incomplete dRTA prevalence has ranged from under 6% to 25% across four cohort studies, despite all using some form of formal urinary acidification testing. Most available data are derived from small, single-center cohorts (typically fewer than 100 patients) or from pooled compilations of published case reports, both of which are subject to selection and publication bias toward more symptomatic presentations. These studies also apply variable diagnostic criteria—ranging from the ammonium chloride loading test considered the gold standard, to the better-tolerated but less sensitive furosemide-fludrocortisone test, to non-standardized criteria based on persistent urinary pH elevation alone—as well as differing case-ascertainment strategies, such as testing only patients with an elevated baseline urine pH versus systematically screening all enrolled patients.

Imaging modality and referral patterns for detecting nephrolithiasis and nephrocalcinosis are similarly inconsistent across studies. As a result, the epidemiological ranges reported earlier in this review should be regarded as approximate, cohort-specific estimates rather than precise, generalizable population prevalence figures, and future studies would benefit from standardized diagnostic protocols to enable meaningful cross-study comparison 7-9, 123

Reversibility of tubular dysfunction appears to be strongly influenced by disease stage. Active inflammatory TIN may respond to immunosuppression, whereas advanced fibrosis is associated with persistent functional impairment. Interstitial fibrosis and tubular atrophy (IFTA) exceeding 50-75% is associated with minimal recovery despite treatment in several series 124. Across immune-mediated kidney diseases, chronic structural damage is a stronger determinant of outcome than residual inflammation 125, 126. In SjD-associated RTA, normalization of urinary acidification is uncommon, and predictors of response remain poorly defined 2.

Biomarkers that distinguish reversible inflammation from irreversible fibrosis are not established. Candidate markers include soluble interleukin-2 receptor (sIL-2R) and urinary tubular injury markers such as kidney injury molecule-1 (KIM-1), neutrophil gelatinase-associated lipocalin (NGAL), and β2-microglobulin 127, 128. These markers reflect injury but have limited specificity for predicting treatment response. Molecular approaches, including urinary microRNA profiling, remain investigational 129.

The absence of randomized trials limits evidence-based treatment selection. Most data are derived from observational studies or extrapolated from other autoimmune kidney diseases. Clinical trial design is complicated by disease rarity and heterogeneity. Strategies such as adaptive trial designs, enriched cohorts, and surrogate endpoints have been proposed, but standardized renal outcomes—including acidification capacity, tubular biomarkers, and kidney function trajectory—are not yet consistently applied 128, 130, 131.

### 7.5 Summary of Evidence Gaps and Future Research Priorities

The current evidence base for SjD-associated RTA is derived almost entirely from observational cohorts, case series, and case reports, and several evidence gaps warrant explicit summary. Epidemiologically, reported prevalence estimates for incomplete dRTA vary widely, ranging from under 6% to 25% across cohort studies (Table 4), a discrepancy that most likely reflects heterogeneous diagnostic criteria and small, single-center study populations rather than true differences in disease burden; standardized, multi-center prospective studies are therefore needed to establish reliable prevalence figures. Mechanistically, key components of the proposed pathophysiological model remain conceptual rather than directly validated: the proximal citrate-handling pathway is extrapolated from general renal physiology rather than confirmed in SjD-specific studies, and the genetic two-hit hypothesis linking transporter gene variants to autoimmune tubular injury has not been directly demonstrated in any clinical study. Diagnostically, candidate biomarkers such as urinary citrate and tubular injury markers, including β2-microglobulin, N-acetyl-β-D-glucosaminidase, kidney injury molecule-1, and neutrophil gelatinase-associated lipocalin, require prospective, multi-center validation before they can be incorporated into routine screening or risk-stratification protocols. Therapeutically, no randomized controlled trials have been conducted in this population, and available treatment data are derived from case series or extrapolated from the management of other autoimmune tubulointerstitial diseases, with no agent yet evaluated against a predefined renal or tubular functional endpoint (Table 3, Section 5.3). Addressing these gaps will require adaptive trial designs, enriched patient cohorts, and standardized renal outcome measures, such as acidification capacity, tubular injury biomarkers, and estimated glomerular filtration rate trajectory, to enable future evidence-based recommendations for this condition.

## 8. Future Directions in Sjögren's Disease-Associated RTA: From Mechanisms to Precision Medicine

Progress in SjD-associated RTA depends on improving early detection, linking mechanisms to measurable phenotypes, and incorporating renal-specific endpoints into clinical studies.

Hypocitraturia may serve as a practical marker for early disease detection. Earlier studies reported hypocitraturia in 74% of patients with primary SjD, and, as noted above, a single-center cohort of 42 patients reported high sensitivity and specificity for identifying dRTA 7, 132; prospective, multi-center validation of this diagnostic performance is needed before urinary citrate can be considered for routine screening. Additional urinary markers, including β₂-microglobulin, α1-microglobulin, and N-acetyl-β-D-glucosaminidase (NAG), reflect tubular injury, while anti-CAII antibodies are associated with impaired urinary acidification 38, 74, 133. Integration of urinary citrate, tubular injury markers, and serological features may facilitate identification of subclinical disease before overt metabolic acidosis develops.

Mechanistic studies need to define how immune-mediated injury translates into specific defects in tubular acidification. Current evidence implicates disruption of CAII, V-ATPase, and AE1, but is largely derived from small series and indirect models 2, 10, 134. High-resolution approaches, including single-cell and spatial transcriptomics, may clarify immune-epithelial interactions and distinguish reversible inflammation from fibrosis, providing a basis for targeted intervention.

Therapeutic development is limited by the absence of renal-specific endpoints in SjD trials. Targeted approaches directed at B-cell activation, B-cell activating factor (BAFF) signaling, and Janus kinase-signal transducer and activator of transcription (JAK-STAT) pathways are supported by mechanistic rationale, but their effects on tubular acidification and long-term renal outcomes remain undefined. Evaluation of these therapies requires endpoints that capture tubular function, including urine acidification capacity, bicarbonate and potassium control, urinary citrate, estimated glomerular filtration rate trajectory, and imaging-based assessment of nephrocalcinosis.

Precision approaches will depend on prospective cohorts and multicenter registries that define disease trajectories and treatment response. Risk models incorporating clinical, biochemical, serological, and genetic variables have identified factors associated with RTA development, including disease duration, autoimmune features, and metabolic abnormalities 135, 136. Integration of these datasets with molecular profiling may further improve patient stratification, although external validation is required before clinical implementation.

## 9. Conclusion

SjD-associated RTA should be understood as an immune-tubular disorder rather than a purely electrolyte abnormality. Disruption of distal acidification reflects immune-mediated injury to tubular epithelial cells and may precede classical sicca symptoms, contributing to frequent diagnostic delay and underrecognition, particularly in subclinical forms such as incomplete dRTA. While alkali therapy corrects metabolic disturbances, treatment response is largely determined by disease stage, with limited reversibility once fibrosis is established. This framework shifts the clinical perspective from descriptive electrolyte disturbance to a mechanism-oriented model of tubular injury, with implications for both diagnosis and therapeutic strategy.

### Key Messages

1. SjD-associated RTA is an immune-tubular disorder rather than a purely electrolyte abnormality, arising from immune-mediated injury to tubular epithelial cells and manifesting predominantly as distal RTA.

2. Renal involvement, including subclinical incomplete dRTA, frequently precedes classical sicca symptoms and contributes to diagnostic delay and underrecognition.

3. An integrated distal-proximal tubular axis, with hypocitraturia as a candidate biomarker, provides a plausible mechanistic link between tubular dysfunction and nephrolithiasis, nephrocalcinosis, and hypokalemic paralysis.

4. Alkali therapy corrects metabolic abnormalities but does not reverse the underlying immune injury; treatment response is stage-dependent, with limited reversibility once fibrosis is established.

5. Early recognition, biomarker-driven risk stratification, and closer nephrology-rheumatology collaboration are needed to enable mechanism-based, precision-guided care.

## Figures and Tables

**Table 1:** Clinical and Biochemical Features of RTA Subtypes in Sjögren's Disease.

Feature	Distal RTA (Type 1)	Proximal RTA (Type 2)	Mixed RTA	Type 4 RTA	Incomplete dRTA
**Relative frequency in SjD**	Predominant phenotype (~80-90%) 3	Rare 1, 20	Occasional 13	Very rare 3	6-25% 7-9 (variable across cohorts)
**Primary defect**	Distal acid secretion failure (α-intercalated cells)	Impaired proximal bicarbonate reabsorption	Combined proximal and distal defects	Aldosterone deficiency or resistance	Partial distal acidification defect
**Serum HCO_3_^-^**	↓	↓	↓	↓	Normal
**Serum K^+^**	↓ (often marked)	↓ (usually mild)	↓	↑	Normal or ↓
**Urine pH**	> 5.5 (inappropriately elevated)	Variable (< 5.5 after steady state)	Variable	Typically <5.5 (may be variable if a secondary or mixed tubular process is present)	>5.5 (after acid load)
**Urine anion gap (UAG)**	Positive	Variable	Variable	Positive (reflecting reduced NH_4_^+^ excretion)	Positive
**FEHCO_3_^-^**	< 5%	> 10-15%	Variable (may be elevated if proximal component present)	< 5%	< 5%
**Key clinical features**	Hypokalemic paralysis, nephrolithiasis, nephrocalcinosis	Fanconi syndrome (phosphaturia, glycosuria)	Overlapping features	Hyperkalemia, mild acidosis	Recurrent stones, nephrocalcinosis
**Pathophysiology in SjD**	TIN with V-ATPase/AE1/CAII dysfunction	Diffuse tubular injury	Extensive tubulopathy	Usually secondary or comorbid	Early or subclinical tubular injury
**Clinical implications**	Dominant phenotype; may represent initial presentation of SjD	Suggests broader proximal tubular involvement	Suggests more extensive disease	Consider evaluation for comorbidities or drug exposure	Often underrecognized; potential target for early detection

Abbreviations: AE1, anion exchanger 1; CAII, carbonic anhydrase II; FEHCO_3_^-^, fractional excretion of bicarbonate; NH_4_^+^, ammonium; RTA, renal tubular acidosis; SjD, Sjögren's disease; TIN, tubulointerstitial nephritis; UAG, urine anion gap; V-ATPase, vacuolar H^+^-ATPase.

**Table 2:** Differential Diagnosis of Distal RTA: SjD-Associated versus Other Autoimmune, Hereditary, and Drug-Induced Causes.

Category	Example Causes	Key Distinguishing Features vs. SjD-Associated dRTA	Diagnostic Clue
SjD-associated dRTA	Autoimmune TIN in primary Sjögren's disease	Sicca symptoms; anti-SSA/Ro, anti-SSB/La positivity; plasma cell-rich TIN on biopsy	Anti-SSA/SSB, biopsy, sicca assessment
Other autoimmune disease	SLE; IgG4-related disease	SLE: anti-dsDNA, hypocomplementemia, multi-organ involvement. IgG4-RD: elevated serum IgG4, storiform fibrosis with IgG4+ plasma cell-rich infiltrate on biopsy 74	Serology (anti-dsDNA, complement, IgG4) and histology
Hereditary dRTA	*SLC4A1* (AE1), *ATP6V1B1*, *ATP6V0A4* mutations	Earlier onset (often childhood); family history; may be associated with sensorineural hearing loss (*ATP6V1B1*); absence of autoimmune serology 59, 78	Genetic testing; family history; audiometry
Drug-induced RTA	Amphotericin B, tenofovir, ifosfamide	Clear temporal relationship to drug exposure; typically reversible after drug withdrawal; absence of autoimmune serology or TIN on biopsy 45, 77	Medication history; resolution after discontinuation

**Table 3:** Evidence for Renal Outcomes and Reversibility of Tubular Dysfunction with Immunosuppressive Agents in SjD-Associated RTA.

Agent	Role in Treatment Hierarchy	Reported Renal/Tubular Outcome	Evidence Level	Key Limitation
Corticosteroids	Most commonly used initial agent, especially during active inflammatory phase	Most consistent reported improvement in tubular function when initiated early	Case series 86-88	No controlled comparison; benefit declines with established fibrosis
Mycophenolate mofetil / Azathioprine	Steroid-sparing, for relapse or incomplete response	Used to maintain control after steroid response; renal-specific outcome data limited	Case series 89	No systematic renal outcome data; efficacy inferred from clinical experience
Hydroxychloroquine	Adjunct for systemic disease control	No direct evidence of improved tubular acidification	90	Not renal-outcome directed
Calcineurin inhibitors	Refractory cases	Reported benefit in small series	Small case series 86	Nephrotoxicity risk; very limited data
Rituximab	Off-label, selected refractory cases	Individual reports of recovery of urinary acidification	Case reports/small series 93	Inconsistent systemic benefit in RCTs; renal tubular outcomes not systematically evaluated 94, 95
TNF-α inhibitors	Investigational	Benefit in individual cases	Case reports 91, 92	Role in SjD-associated RTA remains uncertain

**Table 4:** Reported Prevalence of Incomplete Distal RTA Across Sjögren's Disease Cohorts, with Study-Level Quality Appraisal.

Study	Design	n	Diagnostic Criterion Used	Reported Prevalence	Quality tier	Appraisal note
Both et al., 2015 9(Netherlands)	Prospective, SjD-specific cohort	57	Furosemide-fludrocortisone testing with confirmatory ammonium chloride loading	25% (incomplete dRTA)	High	Prospective design; standardized, validated provocative testing protocol; largest SjD-specific cohort among those cited
Aasarød et al., 2000 123 (Norway)	Retrospective cohort	62	Combined complete + incomplete dRTA; urinary acidification testing	11.3% (combined complete and incomplete dRTA)	Moderate	Older retrospective series; does not separate incomplete from complete dRTA, limiting direct comparability
Jain et al., 2018 8(India)	Prospective cohort	70	Urinary acidification/citrate-based criteria (no confirmatory provocative test)	5.7% (incomplete dRTA alone; 4/70)	Moderate	Prospective design but diagnostic criteria differ from the provocative-testing standard, likely underestimating true prevalence
Coradin et al., 2024 7 (Brazil)	Single-center, retrospective	42	Hypocitraturia-based case definition	4.9% (complete RTA; incomplete dRTA not separately reported)	Low-Moderate	Small single-center sample; biomarker-based rather than provocative-testing-based definition; findings not yet externally validated

Quality tier reflects an informal appraisal (not a formal GRADE assessment) based on study design, sample size, and whether a standardized confirmatory provocative acidification test was used. Estimates from higher-tier cohorts should be weighted more heavily when interpreting the overall prevalence range. Abbreviations: dRTA, distal renal tubular acidosis; SjD, Sjögren's disease.
